# Preparation of Barbed ZnO Fibers and the Selective Adsorption Behavior for BSA

**DOI:** 10.1021/acsomega.1c01454

**Published:** 2021-06-18

**Authors:** Liu Liu, Yuxiang Dai, Yang Qi

**Affiliations:** † Institute of Materials Physics and Chemistry, School of Materials Science and Engineering, Northeastern University, Shenyang, Liaoning 110819, China; ‡ Key Laboratory for Anisotropy and Texture of Materials, Northeastern University, Shenyang, Liaoning 110819, China

## Abstract

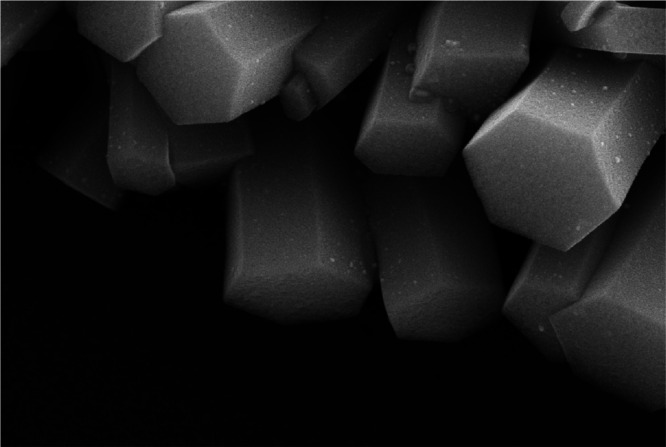

ZnO electrospun nanofibers can act as seed fibers to fabricate multidentate barbed fibers perpendicular to the growth of the fibers using the chemical bath deposition (CBD) method. Fibers with a multirod morphology have a porous grid structure. The sample is easy to recover, and the nonpolar surface in the sample is sufficiently exposed. In the research of barbed fiber fabrication and adsorption on bovine serum albumin (BSA), the effects of different chemical bath conditions on the growth of ZnO nanorods were discussed. Barbed fibers with large slenderness ratios were obtained at a water content of 60 mL at 75 °C. Each milligram of barbed fibers can quickly adsorb about 162 μg of protein within 30 min. The adsorption activity of BSA between polar and nonpolar ZnO surfaces was also studied. The selective adsorption behavior of BSA on the nonpolar surface was revealed.

## 1 Introduction

Serum albumin in bovine serum (BSA) concentrate is a protein that is commonly used in immunodiagnostic procedures, clinical reagents, cell culture media, and protein chemistry in biological laboratories.^1^ The adsorption of proteins on the surface of biological materials is an important step in the basic biological process. The adsorbed protein will further induce subsequent cell landing, diffusion, and other effects.^2,3^ Thus, it is necessary to investigate the adsorption activity of BSA.

With the development of biomedical research, the application of nanomaterials in disease diagnosis,^4^ treatment, cell separation, drug carriers, and nanobiochips has attracted increasing attention. In recent years, zinc oxide used for protein adsorption devices has been used in a variety of biomedical and pharmaceutical fields due to its good biocompatibility,^5^ chemical stability, and ease of preparation.^6,7^ In the research field of adsorption of proteins on ZnO, powder materials have been widely studied.^8,9^ Song et al.^10^ studied the adsorption experiments of GST- and His-labeled recombinant proteins on ZnO. The results showed that ZnO had specific adsorption for new and formed proteins. To characterize the adsorption mechanism between the protein and the material surface, centrifugal treatment was needed to separate the powders from the protein solution. However, it is difficult to recover the powder structure biomaterials with small particle sizes, and there will be some loss in the recovery process. ZnO grown on substrates in the study of protein adsorption has also been widely reported.^11,12^ Limited by the morphology of the sample, the two-dimensional ZnO material grown on the substrate cannot have a high specific surface area. Dorfman et al. demonstrated for the first time that engineered nanoscale ZnO structures can serve as ideal substrates for identifying and screening the protein–protein interaction. Xie et al.^13^ revealed that the ZnO (10-10) surface showed 2 orders of magnitude higher amounts of surface-bound proteins relative to the (0001), (000-1), and (11-20) planes. Wang et al. also found that compared with other morphologies, ZnO nanorods have the best adsorption performance for BSA.

Therefore, we have designed the ZnO material with a porous grid structure composed of ZnO fibers. The research work on ZnO fiber materials has been widely reported.^14^ The electrospun ZnO fibers belong to the polycrystalline wurtzite structure.^15^ However, the polycrystalline fiber mesh is not an idealized adsorbent biomaterial.^16^ In this study, a novel ZnO barbed fiber material has been synthesized. Perpendicularly orientated nanorods grown on ZnO electrospun fibers by an eco-friendly process without involving any hazardous, toxic, or highly corrosive chemical reagents. The morphology of ZnO nanorods was modulated to a certain extent. Barbed fibers not only have multidirectional nanorods but also have better recyclability. By observing the adsorption of BSA, crystal planes with better adsorption performance were summarized, and the direction for further research was provided.

## 2 Results and Discussion

### 2.1 Structural and Morphology Characterizations

**Figure 1:**
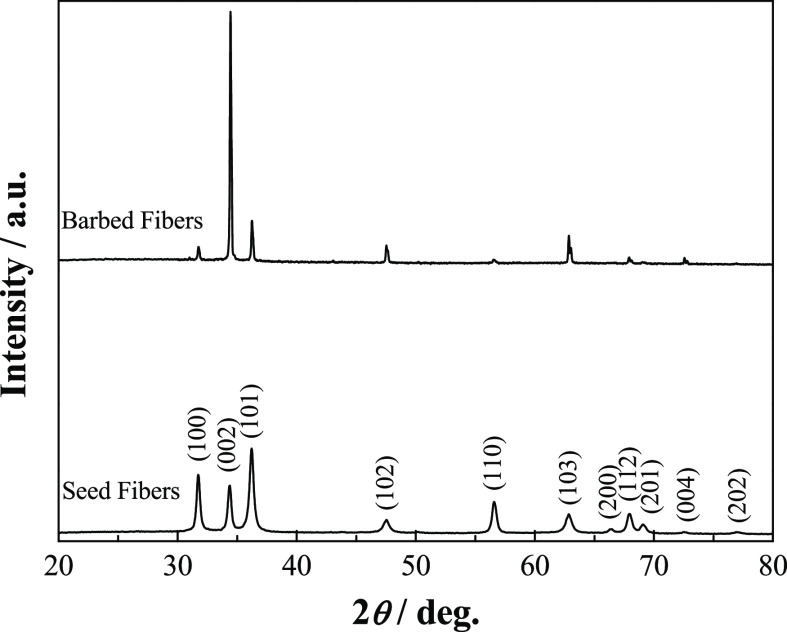
X-ray diffraction patterns of seed fibers and barbed fibers.

Crystallographic properties of seed fibers and barbed fibers samples are shown in Figure 1. The diffraction peaks in the pictures correspond to PDF 36-1451. XRD patterns indicate that both seed fibers and barbed fibers are composed of the wurtzite ZnO structure. In the chemical bath process, the polycrystalline structure of seed fibers provides a large number of nucleation sites. Therefore, a great quantity of ZnO nanorods can be grown on the seed fibers with a small diameter. For crystalline ZnO, the surface energy of the polar surface is relatively low. This is the reason for the preferential orientation of ZnO crystal growth along the *c*-axis. The strongest (002) peak at 34.4° in the pattern of barbed fibers indicates that the hexagonal top surface of the nanorods is the polar surface. Because the nanorods grow perpendicularly to seed fibers, the size limitation of the fiber shape is eliminated, and the chemical bath solution has a high ion concentration. The diffraction pattern with observably smaller full width at half-maximum (FWHM) indicates that barbed fibers have a larger grain size than seed fibers.

**Figure 2:**
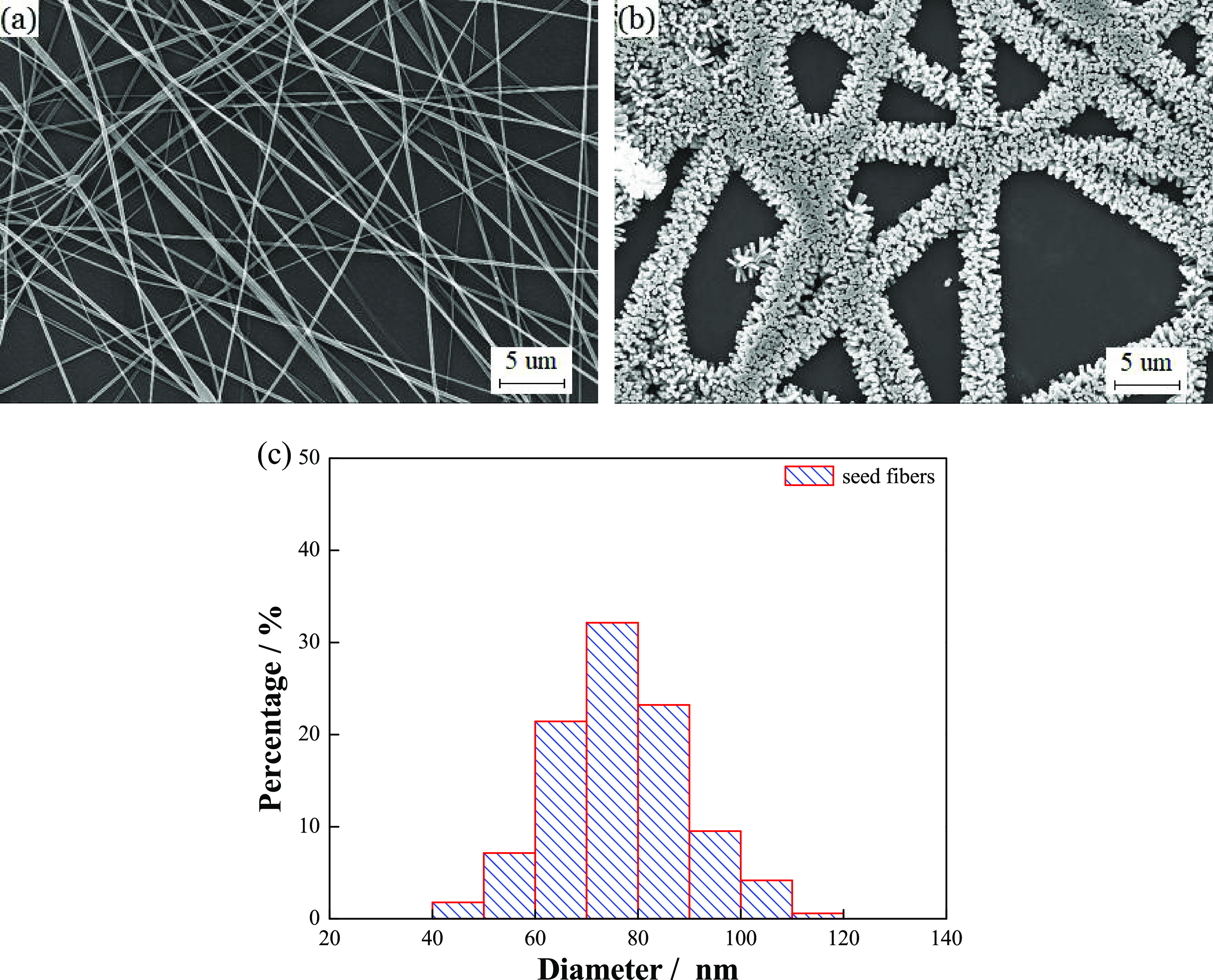
Scanning electron microscopy (SEM) images of (a) seed fibers by electrospinning, (b) barbed fibers prepared after the chemical bath, and (c) diameter statistics of seed fibers.

Figure 2a,b shows the morphology of the seed fibers and barbed fibers. The diameter of the seed fiber is about 80 nm. In the process of chemical bath, a great quantity of hexagonal ZnO nanorods grow from nucleation centers on the seed fibers. After growing the nanorods, the diameter of the barbed fibers reaches a few microns. To reveal the effect of the diameter of seed fibers on the barbed fibers, seed fibers with diameters of 87.6, 81.1, and 76.4 nm were obtained under different spinning voltages of 12.5, 15, and 17.5 kV, respectively. Under the same chemical bath conditions, the diameters of the barbed fibers are 4.17, 4.09, and 4.15 μm. The above phenomenon shows that the diameter of seed fibers within a certain diameter range has little effect on the morphology of the barbed fibers after the chemical bath. This is due to the fact that the size of ZnO nanorods grown on the fibers is much larger than that of seed fibers. After chemical bath deposition (CBD), the diameter of the fiber can reach several microns, while the diameter of the seed fiber is less than 100 nm. The diameter distribution of the seed fiber is shown in Figure 2c. Seed fibers with the polycrystalline structure provide nucleation centers at the initial stage of nanorod growth. The subsequent growth mode of nanorods is mainly influenced by chemical bath conditions.

**Figure 3:**
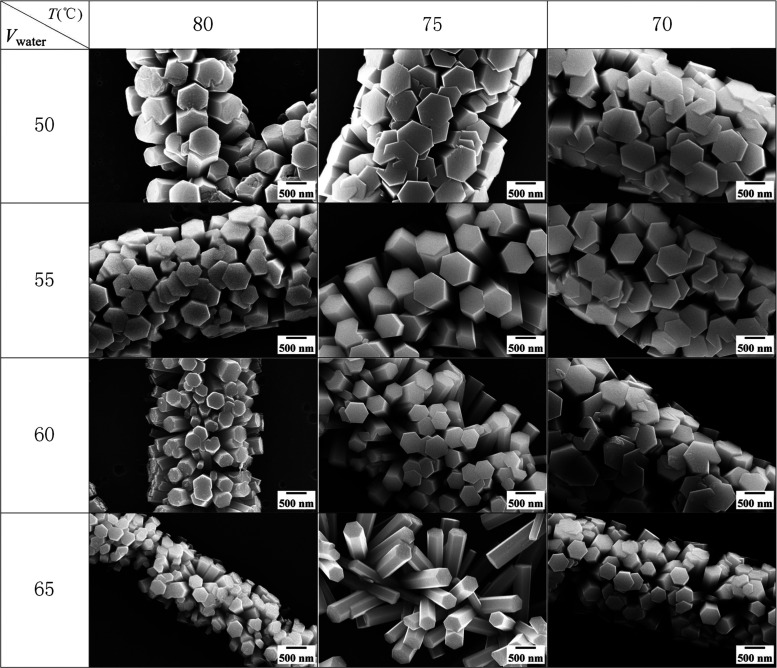
SEM images of barbed fibers in different chemical bath conditions.

Sequentially, the morphology of the samples obtained under different chemical bath conditions has been characterized. The samples of different chemical bath solutions (50, 55, 60, and 65 mL) were observed at bath temperatures of 70, 75, and 80 °C. The diameters of the barbed fibers and nanorods are statistically obtained by Nano Measurer software. The length of nanorods is approximately equal to half the diameter of barbed fibers. For all samples in the control group, the morphologies are shown in Figure 3. Photographs show that less water content in the chemical bath solution corresponds to higher ion concentration, which makes nanorods grow vigorously not only along the *a*/*b*-axis but also along the *c*-axis.

**Figure 4:**
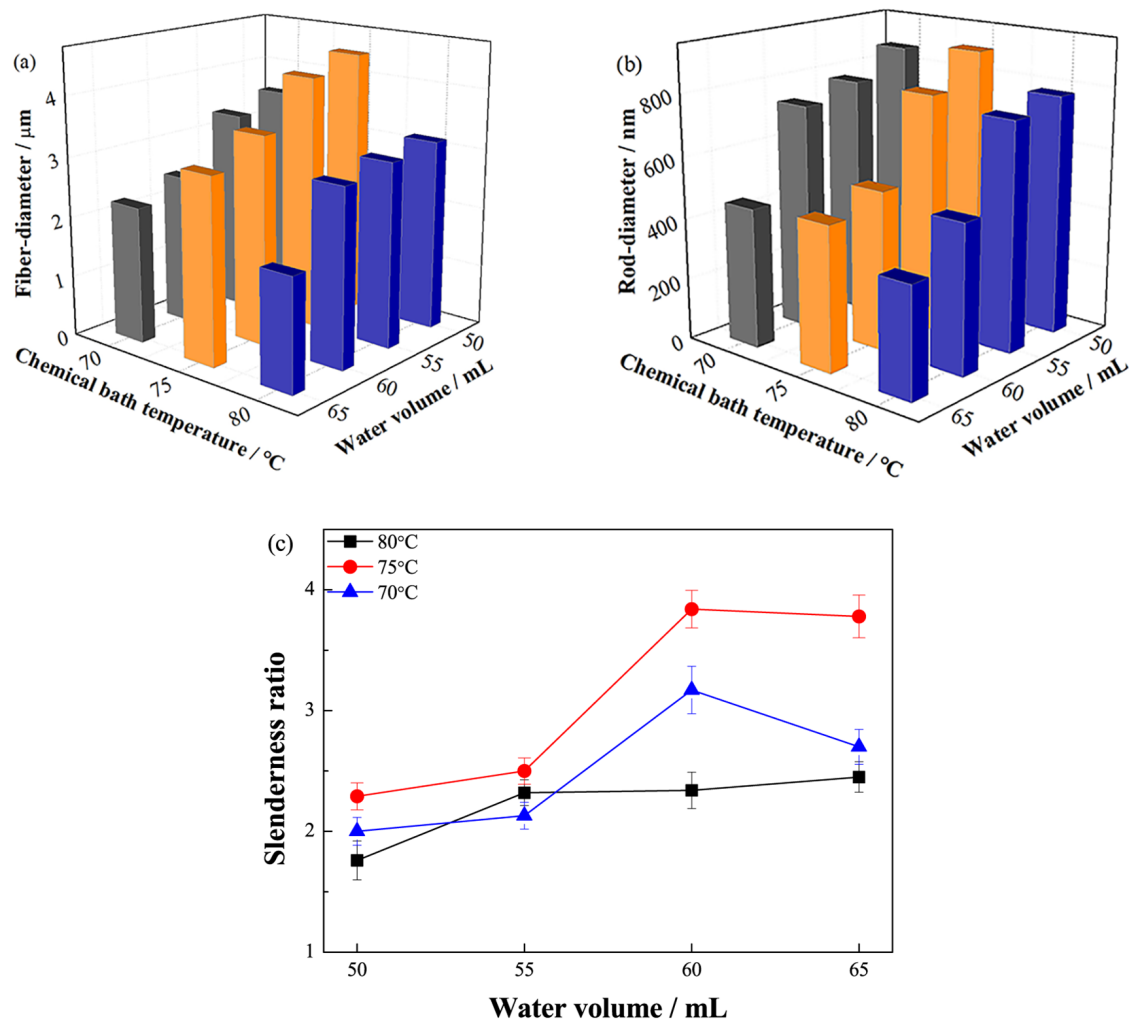
Diameter distribution of (a) barbed fibers and (b) ZnO nanorods, and (c) slenderness ratio of ZnO nanorods.

ZnO nanorods with *c*-axis-preferred orientation have higher surface energy on the nonpolar surface, which is beneficial to the adsorption process. To achieve more nonpolar surfaces, the chemical bath conditions that are more conducive to growth along the *c*-axis are ideal. From Figure 4a, the image clearly illustrates that a larger diameter barbed fiber can always be obtained at 75 °C under each water content condition. This reveals that when seed fibers are chemically bathed at this temperature, the growth conditions of nanorods tend to be more favorable for growth along the *c*-axis. When the degree of supersaturation of the chemical bath solution is low, ZnO is more likely to be heterogeneously nucleated in the buffer solution formed, so the nucleation rate and growth rate of ZnO are slower. In addition, hexamethylenetetramine (HMTA) forms an amine complex with Zn^2+^ in the form of a bidentate ligand, and OH^–^ generated by slow hydrolysis of HMTA reacts with Zn^2+^ ions to form Zn(OH)_2_. Dehydration of Zn(OH)_2_ and hydrolysis of the amine complex form ZnO crystals. Since the hydrolysis rate of the amine compound and HMTA is relatively slow, the morphology of the ZnO nanorods in the uniform and slow reaction is superior to the high concentration chemical bath solution with lower water content. It is obvious in Figure 4b that the chemical bath solution with higher water content has a positive effect on the preparation of nanorods with smaller diameters.

The slenderness ratio of nanorods was calculated by the statistical analysis of the diameter of barbed fibers and nanorods, as shown in Figure 4c. At the optimum temperature, samples with water contents of 60 and 65 mL in the bath solution have larger slenderness ratios. This achieves the goal of optimizing the preparation conditions because the larger slenderness ratio means that more nonpolar surfaces can be obtained. By comparing these two conditions, it is observed that the growth density of nanorods with a water content of 60 mL is higher.

### 2.2 BSA Adsorption Behavior Characterization

**Figure 5:**
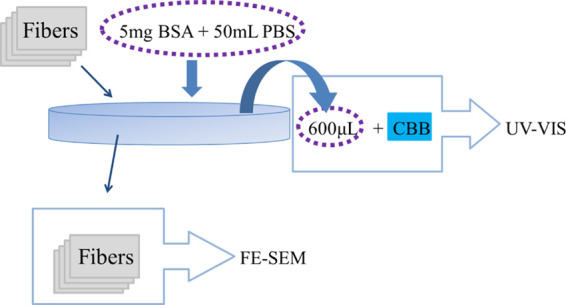
Progress sketch of the BSA adsorbing experiment.

We prepared samples of certain mass for the characterization of protein adsorption properties. Figure 5 shows a schematic diagram of the experimental process for characterizing the protein adsorption properties of the barbed fibers. After removing the fibers, the protein concentration of the unadsorbed BSA solution was used to characterize the adsorption properties of the fibers. The micromorphology of protein-adsorbed barbed fibers was observed to discuss the adsorption mechanism.

**Figure 6:**
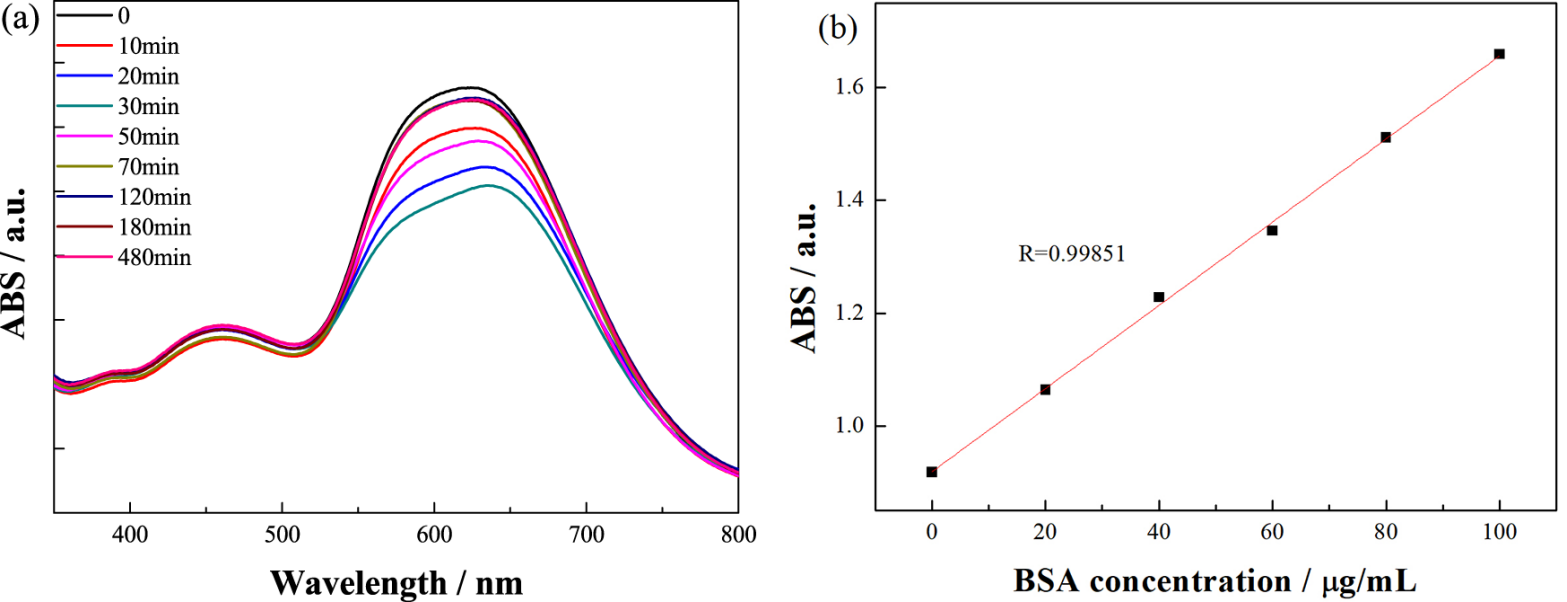
Absorption spectra of the unadsorbed BSA solution (a) according to the standard curve (b).

The absorption spectra of the unadsorbed protein solution are shown in Figure 6a. The light absorption of the unadsorbed BSA protein solution decreased at 595 nm, indicating that the protein content in the solution decreased. The protein concentration in the unadsorbed solution was calibrated according to the standard curve based on the Lambert–Beer law (Figure 6b).

**Figure 7:**
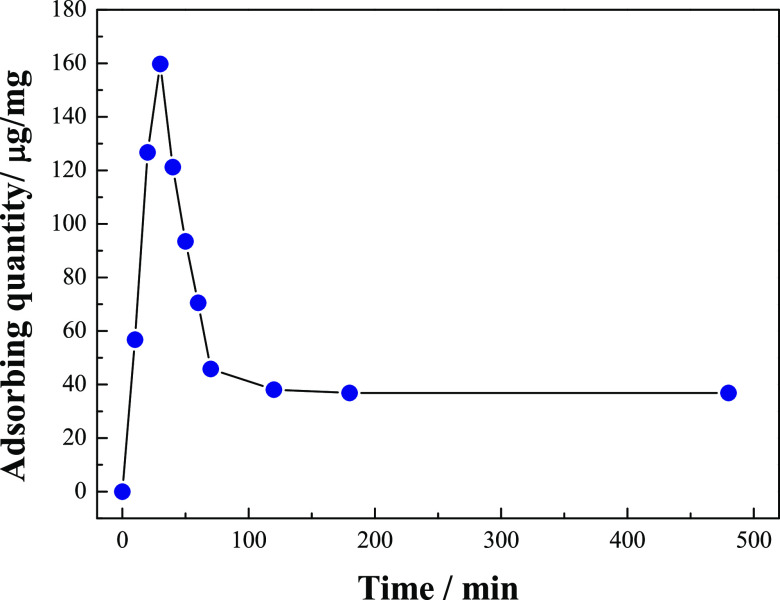
Protein adsorption kinetics curves of BSA on ZnO Barbed Fibers.

At 0.5 h, the light absorption of the protein dyeing solution reached the lowest value, indicating that the protein adsorption capacity of nanorods reached the highest. According to the calculation by formula (1), 40 μg of BSA can be adsorbed stably per milligram barbed fibers. As the adsorption kinetics curve shown in Figure 7, it takes about 2 h for the samples to reach the equilibrium of adsorption and desorption, after which stable adsorption can be achieved. In this process, the maximum adsorption capacity of BSA is 162 μg/mg.

**Figure 8:**
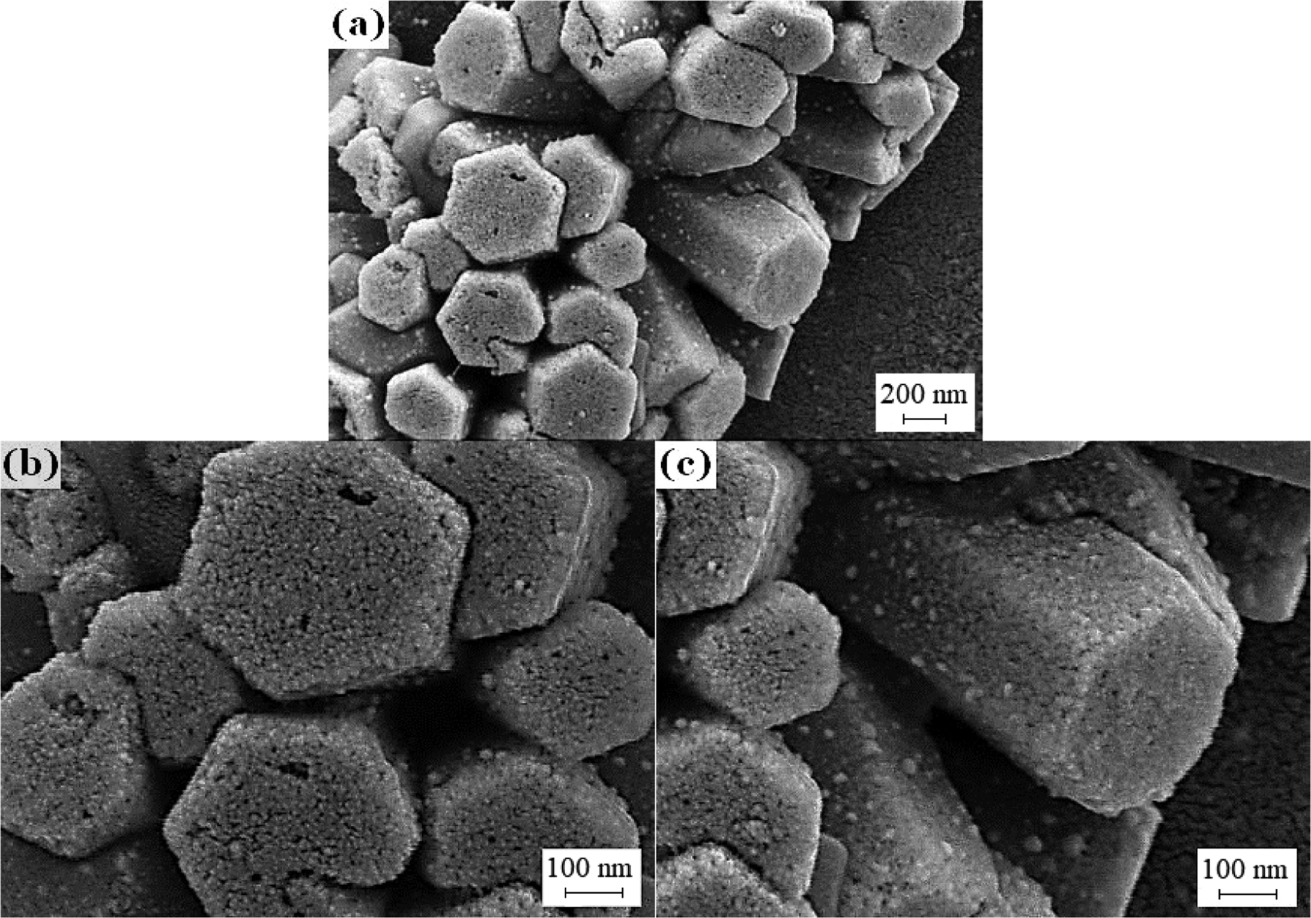
SEM images of (a) barbed fibers with BSA adsorbed, (b) polar planes, and (c) nonpolar planes.

The morphological characteristics of barbed ZnO fibers with BSA are helpful to study the selectivity of protein adsorption. The adsorption forms of BAS on the surface are various. Kozak et al.^17^ studied BSA adsorbed on the surface of hydrogenated and oxidized diamond. For adsorbed BSA, the porous layer morphology and the small spherical nanoparticle morphology were observed by atomic force microscopy (AFM). For the counted BSA clusters, Wang et al.^18^ revealed that the size of BSA was 7.2 ± 0.2 nm, and it was inferred that the white protein substance adsorbed on the barbed fibers should be a single or several protein clusters. Through the SEM photos of a working voltage of 5 kV, Figure 8 can intuitively show that BSA is mostly adsorbed on the nonpolar surface of nanorods. According to the statistics of the number of BSA clusters in the low-magnification SEM photos, the amount of BSA clusters adsorbed by the polar surface of ZnO nanorods is 2.9 × 10^–5^/nm^2^, and the amount of BSA clusters adsorbed on the nonpolar surface is 1.31 × 10^–4^/nm^2^. This confirms that by controlling the conditions of the chemical bath, the preparation of barbed fibers with a larger slenderness ratio of nanorods has achieved the purpose of facilitating protein adsorption.

The adsorption mechanism of BSA on ZnO is considered to be hydrogen bond adsorption^19^ and electrostatic adsorption.^20^ When the pH value of the adsorption environment is 7.4, the electronegativity of the barbed fibers and BSA proteins is opposite due to the different isoelectric points (pl).^21^ ZnO barbed fibers are positively charged because their pl value is larger than the pH value of the adsorption environment. This allows negatively charged proteins to form hydrogen bonds at the lattice oxygen on the nonpolar surfaces of nanorods.^22^

ZnO prepared by the CBD method has been widely reported as a Zn polar surface.^23−26^ During the hydrothermal crystallization of ZnO, the growth of the [0001] direction has been determined to be the fastest under hydrothermal conditions.^27^ This is because the (0001) plane contains a corner of the Zn–O_4_ coordination tetrahedron, which can interact more strongly with the solvated Zn species introduced into the solution, while the (000-1) plane has a coordination tetrahedral face, so there are fewer binding sites. The growth habit of the ZnO crystal is similar to that of the idealized hydrothermal crystal model,^28^ and the fastest growth direction is along [0001]. This may indicate that the existence of [0001]-oriented crystals is determined by the fastest growing direction in hydrothermal conditions. Therefore, the polar surface of nanorods on barbed fibers is most likely to be the termination surface of Zn.^29^ The hydroxide layer is composed of OH^–^, which is difficult to adsorb proteins on it.^30^ In addition to hydrogen bonding and electrostatic adsorption, Rezek et al. studied the adhesion of BSA to the ZnO surface by atomic force microscopy and atomic-scale computing by the force-field method. The AFM observations were corroborated by atomic-scale simulations of BSA on the (0001) ZnO surface using the force-field method and showing rearrangements of Zn surface atoms.^31^

Table 1 shows the adsorption properties of BSA on ZnO nanomaterials with different morphologies. Except for ZnO hollow spheres (ZnO HSs), barded fibers have better adsorption properties compared with powder morphology materials. It is noteworthy that the maximum adsorption capacity of barbed fibers in the adsorption process is higher than that of other types of nanomaterials. This phenomenon may be explained by the morphology of the barbed fibers. Compared with nanoarrays, thin films, and randomly oriented powder materials, the nonpolar surface with adsorption advantages is more fully exposed. This allows the barbed fibers to quickly adsorb a large amount of BSA. Hansda et al.^32^ confirmed that the alternate multilayer growth of the ZnO/BSA layer-by-layer film could be deposited via the electrostatic interactions, and the BSA molecular domains adsorbed on the ZnO surface may have the self-association effect. Therefore, we speculate that due to the bulk effect of a large amount of BSA, the protein on the ZnO surface may be desorbed in avalanche style. This provides a feasible condition for the research and development of new applications in the biomedical field. In addition, the doping of transition metal ions or irradiation modification to the barbed fibers will also be a topic for further in-depth research.

**Table 1:** Adsorption Performance of BSA by ZnO with Different Morphologies

material	*Q*_max_ (μg/mg)	*Q*_ad_ (μg/mg)
ZnO hollow sphere^33^		80
ZnO nanorods^34^	40	38
ZnO nanosheets^35^	48	39
ZnO nanobeams^35^	40	30
this work	162	40

## 3 Conclusions

*C*-axis-oriented ZnO nanorods were grown on electrospun nanofibers by chemical bath deposition. A composite barbed fiber with a grid structure was obtained. Barbed fibers are easier to recycle than powder materials. Compared with nanorod arrays, the (100) planes are more sufficiently exposed in the protein solution environment and can grow into a multilayer grid structure. To prepare more nonpolar planes, the length and diameter of nanorods were controlled by modulating chemical bath conditions. The optimum chemical bath conditions were a water content of 60 mL at 75 °C. The results of adsorption experiments show that BSA has the selective adsorption behavior on the nonpolar surface due to the different surface suspension bonds between the nonpolar surface and the Zn-termination polar surface. Therefore, the (100) planes of the ZnO nanorods have the optimum adsorption capacity for the BSA protein. The maximum adsorption capacity of BSA on barbed fibers is 162 μg/mg and finally stabilized at 40 μg/mg.

## 4 Experiments and Characterization

### 4.1 Chemicals

Poly(vinyl alcohol) (PVA, 1750 ± 50; M = 80000), zinc acetate (Zn(CH_3_COO)_2_·2H_2_O), hexamethylenetetramine (HMTA), Coomassie Brilliant Blue G-250, phosphoric acid (H_3_PO_4_), BSA protein powder, and phosphate-buffered saline (PBS; pH 7.4; Na_2_HPO_4_, KH_2_PO_4_, NaCl, KCl) were purchased from Macklin Reagent. All chemicals were of analytical grade and required no further purification, and all aqueous solutions were prepared with distilled water.

### 4.2 Preparation of Seed Fibers

In the course of the experiment, the precursor sol for electrospinning was prepared. PVA was used as a template for the as-spun precursor fibers before sintering. The detailed preparation process of the precursor sol was as follows. An 8 wt % PVA aqueous solution has been obtained by dissolving the swelled PVA aqueous solution at 93 °C for 5 h. After that, 10 mL of a 23% Zn(CH_3_COO)_2_·2H_2_O aqueous solution was added into 50 g of the PVA aqueous solution at a slow dropping rate. The precursor solution was stirred at 60 °C for at least 2 h. Finally, the whole precursor solution was aged for 24 h at room temperature.

The precursor fluid was electrospun into a fiber shape through a lab-made electrospinning system. In the course of the electrospinning process, the deposition distance between the collecting plate (connecting ground) and the syringe needle (high voltage terminal) was kept at 10 cm. The needle type and the environmental conditions (temperature and humidity) were kept constant. According to our previous work,^36^ 17.5 kV was the optimal spinning voltage among the voltage gradient (7.5–20 kV) in the experiment. After obtaining the PVA and Zn(CH_3_COO)_2_ as-spun fibers under optimized voltage with a 200 μL/h dosing rate, the precursor fibrous sample was subjected to the heat treatment process to sinter the ZnO crystals and eliminate the organic polymer. To remove the polymer template, the as-spun fibers were sintered in a well furnace at 447 °C for 5 h. After the furnace cooled, ZnO polycrystalline seed fibers were prepared.

### 4.3 Preparation of Barbed Fibers

The obtained seed fibers were subjected to a chemical bath under certain conditions. The solution used for CBD was prepared as follows: to make the solution containing the same molar mass of zinc ions and hydroxyl ions, we dissolved 0.2744 g of Zn(CH_3_COO)_2_ and 0.1752 g of HMTA in a certain amount of deionized water. The bath solution was obtained without stirring at room temperature. We immersed the sample upward in the chemical bath for 4 h at different temperatures by a water bath pot with a condensation tube. In the process of preparing barbed fibers by the CBD method, zinc acetate dihydrate and HMTA were added to water for reaction. HMTA was used as buffer and reactant here because of its slow and continuous hydrolysis in aqueous solution. This provided the suitable pH range and sufficient OH for ZnO deposition so that the reaction proceeded gently and smoothly. The whole reaction process is as follows^37^











### 4.4 Protein Adsorption Quantity Measurement

To characterize the adsorption properties of BSA on nanorods, static adsorption experiments were carried out. Twenty milligrams of barbed fibers were immersed in 50 mL of a 100 μg/mL BSA PBS solution. After adsorption, the concentration of the unadsorbed protein solution was estimated using the Bradford assay.^38^ Six hundred microliters of the protein solution was taken and stained with 3 mL of Coomassie Brilliant Blue G-250. Absorbance detection was performed on the staining solution within 5 min to determine the protein concentration. Adsorption (*Q*_ad_) was calculated according to the following formula
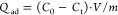
1In the formula above, *C*_0_ is the initial concentration of the BSA solution (μg/mL), *C*_t_ is the concentration of the BSA solution (μg/mL), *V* is the volume of the BSA solution (mL), and *m* is the mass of the barbed fiber sample (mg). The Petri dish was stationary during the adsorption experiment at room temperature.

### 4.5 Analytical Apparatus

The morphological characteristics of fibers were observed using a field emission scanning electron microscope (FESEM, Zeiss Extra Plus, operating voltage of 15.0 kV), and the crystalline structure of the electrospun fibers was characterized by X-ray powder diffraction (XRD, Rigaku D/max diffractometer with Cu Kα radiation λ = 1.5405 Å). Moreover, an ultraviolet–visible absorption spectrophotometer (UV–vis DRS, HITACHI U-3900) was used for analyzing the remaining solution protein concentration according to the predrawn standard curve.
